# Multimodal imaging of laser-induced choroidal neovascularization in pigmented rabbits

**DOI:** 10.1038/s41598-023-35394-z

**Published:** 2023-05-24

**Authors:** Van Phuc Nguyen, Jessica Henry, Josh Zhe, Justin Hu, Xueding Wang, Yannis M. Paulus

**Affiliations:** 1 Kellogg Eye Center, Department of Ophthalmology and Visual Sciences, University of Michigan, 1000 Wall Street, Ann Arbor, MI 48105 USA; 2 Department of Biomedical Engineering, University of Michigan, Ann Arbor, MI 48105 USA

**Keywords:** Biomedical engineering, Macular degeneration

## Abstract

This study aimed to demonstrate longitudinal multimodal imaging of laser photocoagulation-induced choroidal neovascularization (CNV) in pigmented rabbits. Six Dutch Belted pigmented rabbits were treated with 12 laser lesions in each eye at a power of 300 mW with an aerial diameter spot size of 500 μm and pulse duration of 100 ms. CNV progression was monitored over a period of 4 months using different imaging techniques including color fundus photography, fluorescein angiography (FA), photoacoustic microscopy (PAM), and optical coherence tomography (OCT). All treated eyes developed CNV with a success rate of 100%. The margin and morphology of CNV were detected and rendered in three dimensions using PAM and OCT. The CNV was further distinguished from the surrounding melanin and choroidal vessels using FDA-approved indocyanine green dye-enhanced PAM imaging. By obtaining PAM at 700 nm, the location and density of CNV were identified, and the induced PA signal increased up to 59 times. Immunohistochemistry with smooth muscle alpha-actin (αSMA) antibody confirmed the development of CNV. Laser photocoagulation demonstrates a great method to create CNV in pigmented rabbits. The CNV was stable for up to 4 months, and the CNV area was measured from FA images similar to PAM and OCT results. In addition, this study demonstrates that contrast agent-enhanced PAM imaging allows for precise visualization and evaluation of the formation of new blood vessels in a clinically-relevant animal model of CNV. This laser-induced CNV model can provide a unique technique for longitudinal studies of CNV pathogenesis that can be imaged with multimodal imaging.

## Introduction

Age related macular degeneration (AMD) is a leading cause of visual impairment and blindness in humans in developed countries^1–5^. There are two major types of AMD: neovascular or wet AMD and dry AMD. Dry AMD is associated with the deposit of drusen and geographic atrophy of the retinal pigment epithelium (RPE), choriocapillaris, and photoreceptors. Wet AMD is characterized by the development of choroidal neovascularization (CNV)^6^. The growth of new blood vessels in CNV is a pathological process in which new blood vessels grow from the choroidal vasculature into the subretinal space, leading to the formation of a neovascular membrane. The new vessels are fragile and prone to bleeding and leakage, which can lead to the accumulation of fluid and blood in the subretinal space. Rupture of these new vessels can result in hemorrhage, fibrosis, and leakage, further exacerbating the damage to the retina and ultimately causing irreversible vision loss^7–9^. CNV is a common complication of several ocular diseases, including age-related macular degeneration (AMD), myopia, and ocular inflammation. The exact mechanism of CNV development remains an area of active investigation. Approximately 10% of patients with dry AMD will develop wet AMD.

Many animal models have been created to mimic CNV development in humans. Most commonly, these models use either subretinal injections of vascular endothelial growth factor (VEGF) and Matrigel or laser burns to induce CNV. The former method attempts to mimic increased VEGF expression as seen in AMD and promote angiogenesis^10–12^. Previous studies have shown subretinal injections yield a lower success rate in CNV as compared to laser photocoagulation. Previous studies found a 33% success rate of creating CNV with VEGF injections and a 70% success rate using laser photocoagulation in mice^1,13^. In a previous study, our group has shown a high success rate of the CNV model using OCT-guided subretinal injection of VEGF and Matrigel^10,14–16^. Although CNV developed after injection, there were several complications and side effects which occurred during subretinal injections, including photoreceptor degeneration, degeneration of choroidal vessels, cataract, and retinal neovascularization^12^. Laser-induced CNV is a method that can reduce these complications. Currently, mouse^17^, primate^18^, and rat^19^ models of laser-induced CNV exist. To date, no CNV models of laser photocoagulation demonstrating CNV have been successful in pigmented rabbits, although the use of Rose Bengal to create photothrombotic retinal vein occlusion and secondary CNV has been described in White New Zealand rabbits^15,16,20,21^. Although mouse models might be cost-efficient, the eyes are significantly smaller than human eyes and have numerous physiological differences from human eyes^22^. The eyeball size of the mice is estimated to be 8 times smaller than that of the human eye^23,24^. The miniature nature of mouse eyes also makes it difficult to aim precise laser burns^22^. In contrast, rabbit eyes have numerous physiological and anatomical similarities to human eyes, making them good animals for clinical translation. Rabbit eyes have an average axial length of 18 mm as compared to an average of 23 mm for human eyes^25^. However, both mice and rabbits do not have a fovea, and the CNV angiogenesis seen in the laser photocoagulation model fundamentally differs from the genetically influenced, chronic, elderly pathology of AMD^17,26,27^. The animal retinal environment is healthy, and the resultant angiogenesis appears as a result of the inflammation and trauma caused by laser treatment rather than AMD caused by aging or genetic/environmental factors as seen in humans.

In order to follow CNV development, several imaging modalities like fluorescein angiography (FA), optical coherence tomography (OCT), OCT angiography (OCTA), scanning laser ophthalmoscopy (SLO), and photoacoustic microscopy (PAM) have been widely used in rodents (mice and rats)^28–31^ and large animals (rabbits)^10,15,16,20,32–34^. Each type of imaging modality has its own benefits and drawbacks, but together they allow for high-resolution and longitudinal tracking of CNV development in animal models. Spectral domain OCT (SD-OCT) imaging is non-invasive and gives a high-resolution image of retinal features including the internal limiting membrane, ganglion cell layer, retinal nerve fiber layer, inner plexiform layer, inner nuclear layer, outer plexiform layer, outer nuclear layer, external limiting membrane, photoreceptor layer, ellipsoid zone, retinal pigment epithelium, choriocapillaris, choroid, and newly developed choroidal neovascularization (CNV) in pathologic states. However, it has limited ability to visualize deeper choroidal structures in pigmented retina^35^. In contrast, PAM has the potential to improve visualization of the deeper choroid^14–16,20,30,35–37^. PAM is a non-invasive, non-ionizing technique that utilizes the conversion of light energy to sound. This occurs due to a laser pulse-induced thermal expansion of tissue, which creates acoustic waves that can then be detected with an ultrasonic probe to visualize deeper retinal and choroidal structures^20,35^. Combining OCT and PAM data allows for a completely non-invasive technique to track CNV development with high resolution. Shah et al. have described a multimodal PAM, OCT, and SLO system to visualize laser-induced CNV in mice and rats with high resolution^22^. Our group has developed a multimodal PAM and OCT imaging system that can differentiate CNV in larger animals (rabbits) with great contrast and high resolution^20^. The uniqueness of our system relies on the low excitation laser energy (~ 80 nJ), which is about half below the ANSI safety limit (~ 160 nJ at 578 nm)^14^. Our novel multimodal imaging system can provide a high spatial resolution of 4.1 μm and 3.8 μm for PAM and OCT, respectively, as described previously^38^. The acquisition time is 65 s to achieve three-dimensional imaging with a scanning resolution of 256 × 256 pixels.

This research aims to create an efficient and reliable method to induce CNV in pigmented Dutch Belted rabbits with less side effects to the surrounding retinal tissues using millisecond retinal photocoagulation laser without the use of Rose Bengal or creation of a retinal vein occlusion. This novel, robust rabbit model will be monitored with high resolution and depth with multimodal PAM, OCT, and FA imaging for stimulating translational AMD research. Pigmented rabbits share many anatomical, physiologic, and biochemical features with human eyes to allow for clinical and human translational potential.

## Results

### In vivo fluorescent imaging of CNV

**Figure 1:**
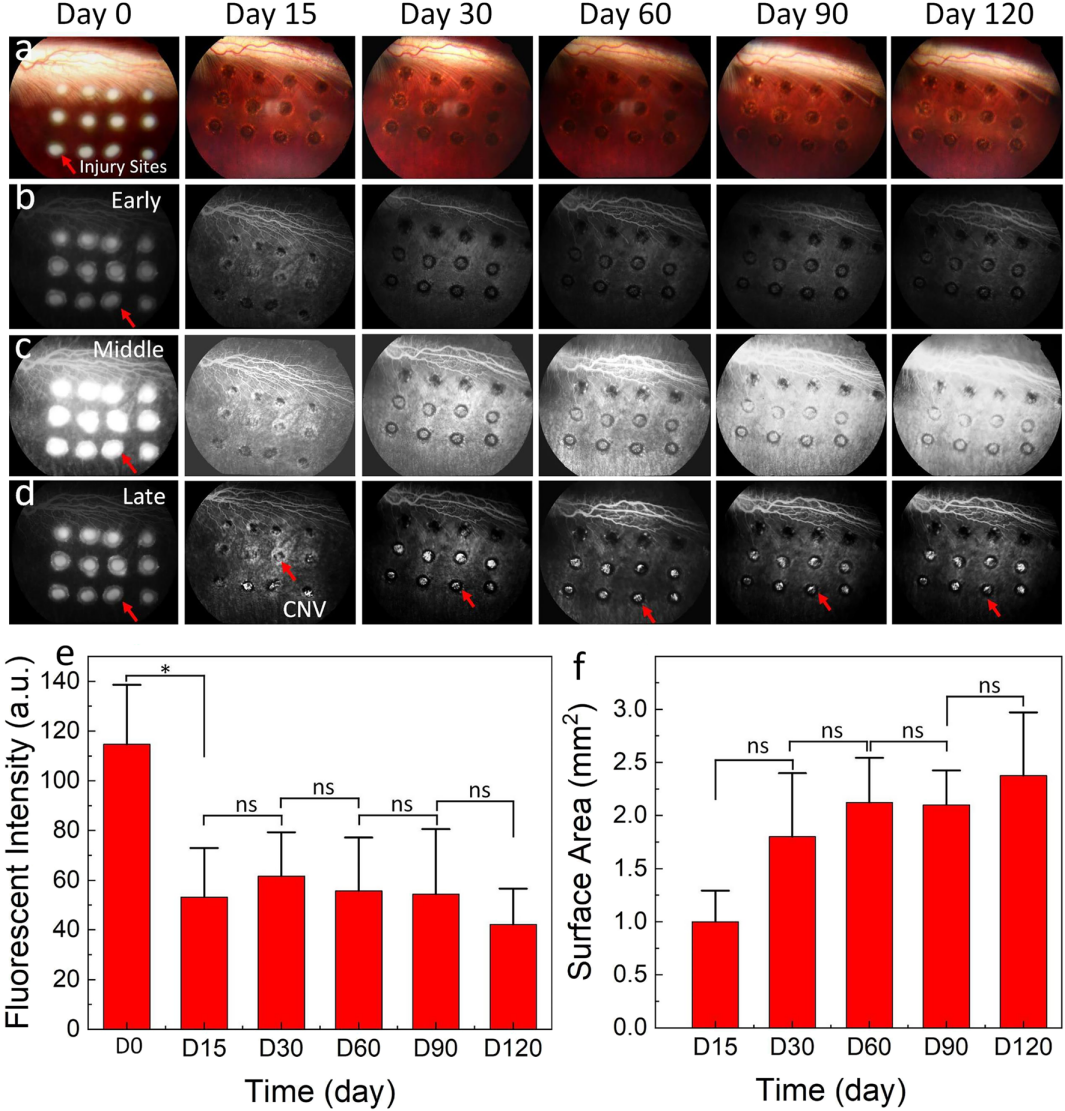
Color photograph and fluorescein angiography imaging of CNV: (**a**) Photograph of the fundus obtained immediately after laser photocoagulation (Day 0) and followed up at days 15, 30, 60, 90, and 120. The laser lesions were clearly visualized after laser illumination (red arrow). Strong backscattering of the light (white areas) indicated the damaged RPE after treatment. Scar tissues were detected on the treated animal from days 15 to days 120 post-treatment. At the laser lesion sites, the tissues changed to dark, and new tissues were observed within the laser-injured area. No evidence of retinal tortuosity was found on the color images, illustrating that the laser only disrupted the targeted area without affecting the adjacent tissues. (**b**–**d**) Fluorescein angiography (FA) is acquired at different stages: early, middle, and late phases. These images provide morphology of retinal vessels, capillaries, laser injury sites, and CNV lesions. CNV leakage was detected at late phase FA from day 15–120. (**e**) A graph of the fluorescent intensity measured at different time points: day 0, 15, 30, 60, 90, and 120 (n = 3, *p* < 0.001). (**f**) Quantification of surface area. Error bars shown as mean and standard deviation.

All of the rabbit fundi were imaged immediately after laser treatment and at days 15, 30, 60, 90, and 120 using color fundus photography and fluorescein angiography (FA). As shown in Fig. 1, the retinal vessels, nerve fibers, and laser lesions were clearly visible on the color fundus image immediately after laser treatment. At the laser-injured sites (Fig. 1a), the retinal tissues changed from red to white with the tissue photocoagulation with visible damage and/or rupture to Bruch’s membrane. This result was confirmed by FA images (Fig. 1b–d). The vascular networks and laser lesions were detected in the mid-phase FA images (Fig. 1c). The late fluorescent leakage revealed the treated areas with FA hyperfluorescence that progressively enlarges over time with hazy borders consistent with CNV leakage. Late-phase FA images allowed for visualization of fluorescein leakage at the location of laser-injured sites post-laser treatment and newly developed CNV (Fig. 1d). The fluorescent leakage was observed immediately after laser photocoagulation as a result of laser-ruptured choroidal vessels. But the leakage of the fluorescent dye from the vessels was observed at the center of laser lesions on day 15 on late phase FA images due to the small molecules of fluorescent dye extravasated at the CNV, confirming the progression of angiogenesis. We also detected the fluorescent leakage within the treated areas on day 120. This illustrated that CNV stabilized over time. By segmentation to isolate the margin of CNV area for measurement of fluorescent intensity and surface area, we found that the highest fluorescent intensity was achieved at day 0 (immediately post laser treatment) as shown in Fig. 1e–f. Then, the fluorescent signal was reduced about 46% from 114.68 ± 23.96 (a.u.) at day 0 and 53.20 ± 19.74 (a.u.) at day 15, likely due to the healing of the damaged choroidal vessels and changes to the angiogenesis. The fluorescence intensities were slightly decreased by about 21% at day 120 (fluorescent intensity = 42.22 ± 14.38 (a.u.)) with a reduction in the angiogenesis. The area of the angiogenesis gradually increased from day 15 when the neovascularization was detected (Area = 1.00 ± 0.29 mm^2^) and reached a peak area of 2.38 ± 0.60 mm^2^ at day 120.

### In vivo PAM and OCT imaging of CNV

**Figure 2:**
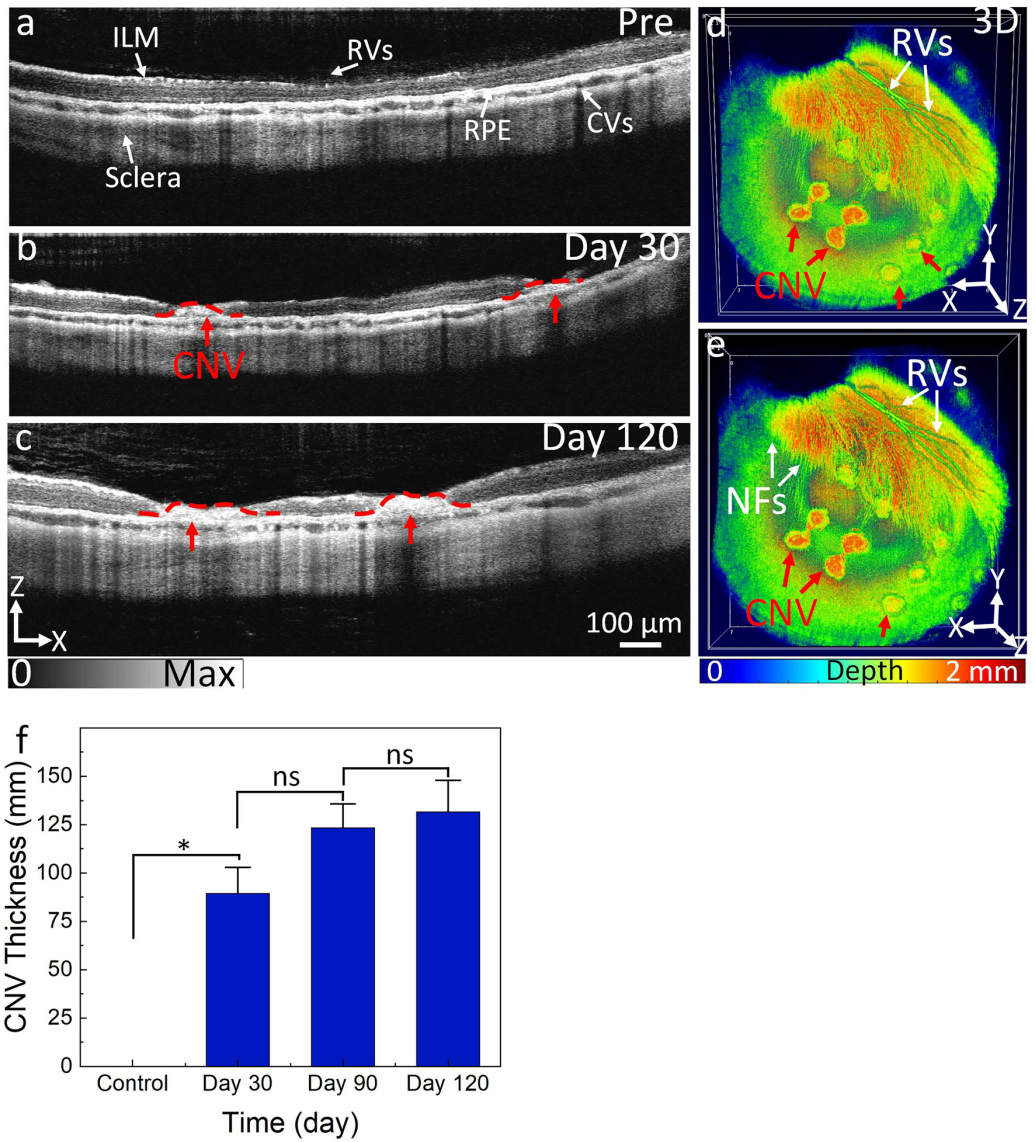
In vivo OCT imaging of laser-induced CNV in rabbits: (**a**) 2D OCT image obtained before laser treatment. Retinal structures such as internal limiting membrane (ILM), retinal vessels (RVs), choroidal vessels (CVs), retinal pigment epithelium (RPE), and sclera. (**b**–**c**) OCT image obtained after laser treatment at days 30 and 120. CNV lesion was developed in the subretinal space (red dotted line). (**d**–**e**) 3D OCT volumetric visualization was achieved on days 30 and 120, respectively. The *en face* 3D OCT clearly shows the location of CNV (red arrows), retinal vessels, and nerve fiber layer. (**f**) A bar graph measuring CNV thickness. Data present as mean and standard deviation (n = 3, *p* < 0.005).

**Figure 3:**
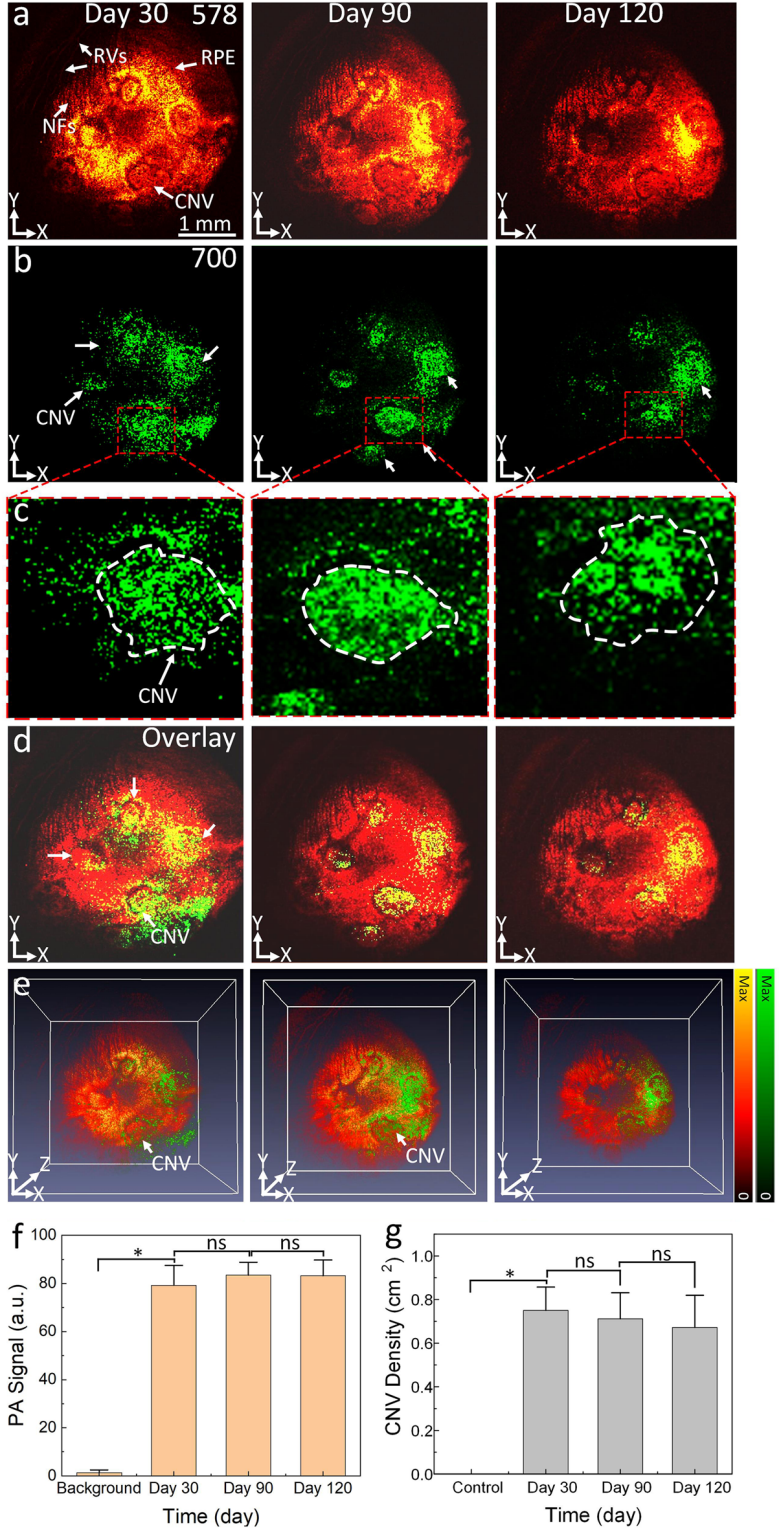
In vivo PAM visualization of CNV lesions: (**a**–**b**) 2D PAM images achieved using different wavelengths of 578 nm (**a**) and 700 nm (**b**) at different time points: day 30, 90, and 120. Retinal vessels (RVs), nerve fibers (NFs), RPE, and formed CNV were observed with high contrast and quality using 578 nm. Note that CNV lesions were differentiated on the PAM images obtained at 700 nm at 15 min after intravenous injection of ICG dye due to the extravasation of ICG at CNV. (**c**) Magnification of the margin of CNV. White dotted circle indicates the boundary of CNV. Pseudo-green color shows the margin of CNV. (**d**) Co-registered PAM images. (**e**) 3D overlay images. (**f**) Quantification of PA signals obtained at 700 nm. (**g**) A graph of CNV density. Data shows as mean and standard deviation (n = 3, *p* < 0.005).

The progression of CNV after laser treatment was monitored longitudinally using non-invasive, high-resolution SD-OCT imaging for up to 120 days (Fig. 2). Internal limiting membrane (ILM), retinal vessels (RVs), choroidal vessels (CVs), sclera, ellipsoid zone (EZ), and retinal pigment epithelium (RPE) layers were detected in OCT images before laser treatment (Fig. 2a). At day 28 post laser treatment, CNV was developed around laser lesions (Fig. 2b). The progression in size, structure, and CNV area was stable for up to 120 days (Fig. 2c). CNV evidence was observed in the ONL, EZ, and inner choroid. There was no change observed in the adjacent retinal tissues nor was vessel formation detected in those areas. The CNV lesion’s structure, RVs, and nerve fiber layer (NFL) were clearly visualized on 3D OCT images (Fig. 2d–e). Depth-encoded 3D OCT images also illustrated that CNV lesions localized to a deeper plane than RVs and NFL. By segmentation to isolate the newly developed CNV, the thickness of the CNV was measured and determined to be about 89.34 ± 13.53 mm at day 30 post-injection using ImageJ software. The CNV thickness kept growing and increased by 40% at day 90. Then, the CNV was almost stable from day 90 to day 120 (CNV thickness = 123.44 ± 12.20 mm for day 90 vs. 131.64 ± 16.36 mm for day 120). This illustrated that CNV became stable over a period of 3 months. To improve discernment of CNV from the surrounding microvasculature, we performed PAM imaging on the rabbit after laser photocoagulation at day 30, 90, and 120 (Fig. 3). In these experiments, PAM images were obtained at two different excitation wavelength of 578 nm and 700 nm after intravenous injection of indocyanine green (ICG) dye. The acquired *en face* PAM image provides comprehensive details of retinal vessel morphology, RPE, NFL, RVs, and CNV. As shown in these PAM images, CNV grew inside the laser photocoagulation lesions. Although the CNV at the center and the border of the lesion could be barely visualized due to the different absorption contrast, it is difficult to differentiate between CNV, RPE, and the new growth of angiogenic capillaries. On the other hand, the margin of CNV was clearly observed on the PAM images obtained at 700 nm post injection of ICG (Fig. 3b–c). The high PA signal allows one to distinguish CNV against the surrounding retinal vessels, a consequence of strong absorption of ICG while hemoglobin has relatively low absorption at 700 nm. Co-registration of PAM images obtained at 578 and 700 nm on the same orthogonal imaging planes shows the degree of CNV growth and allows one to distinguish CNV from the adjacent microvasculature and RPE (Fig. 3d–e, and supplementary video 1). This demonstrates the potential of PAM imaging for the diagnosis of CNV.

By quantification of the PA signal isolated from the CNV obtained at 700 nm and CNV density (CNVD) at different time points: day 30, 90, and 120 (Fig. 3f–g), the PA signal was increased by 59-fold at day 30 from 1.34 ± 0.07 (a.u.) for background to 79.25 ± 8.24 (a.u.) at 30 days (n = 3, *p* < 0.01). Then, the PA signal was not significantly changed from days 90 to 120 (PA_Signal_ = 83.46 ± 5.33 (a.u.) for 90 days and 83.17 ± 6.60 (a.u.) for 120 days, n = 3, *p* > 0.05). Vessel density also increased rapidly to peak at 0.75 ± 0.11 cm at 30 days. The CNVD then gradually decreased by 11% by day 120 (CNVD = 0.67 ± 0.15 cm). However, no statistical difference was observed between these time points (n = 3, *p* > 0.05).

### Histological analysis

**Figure 4:**
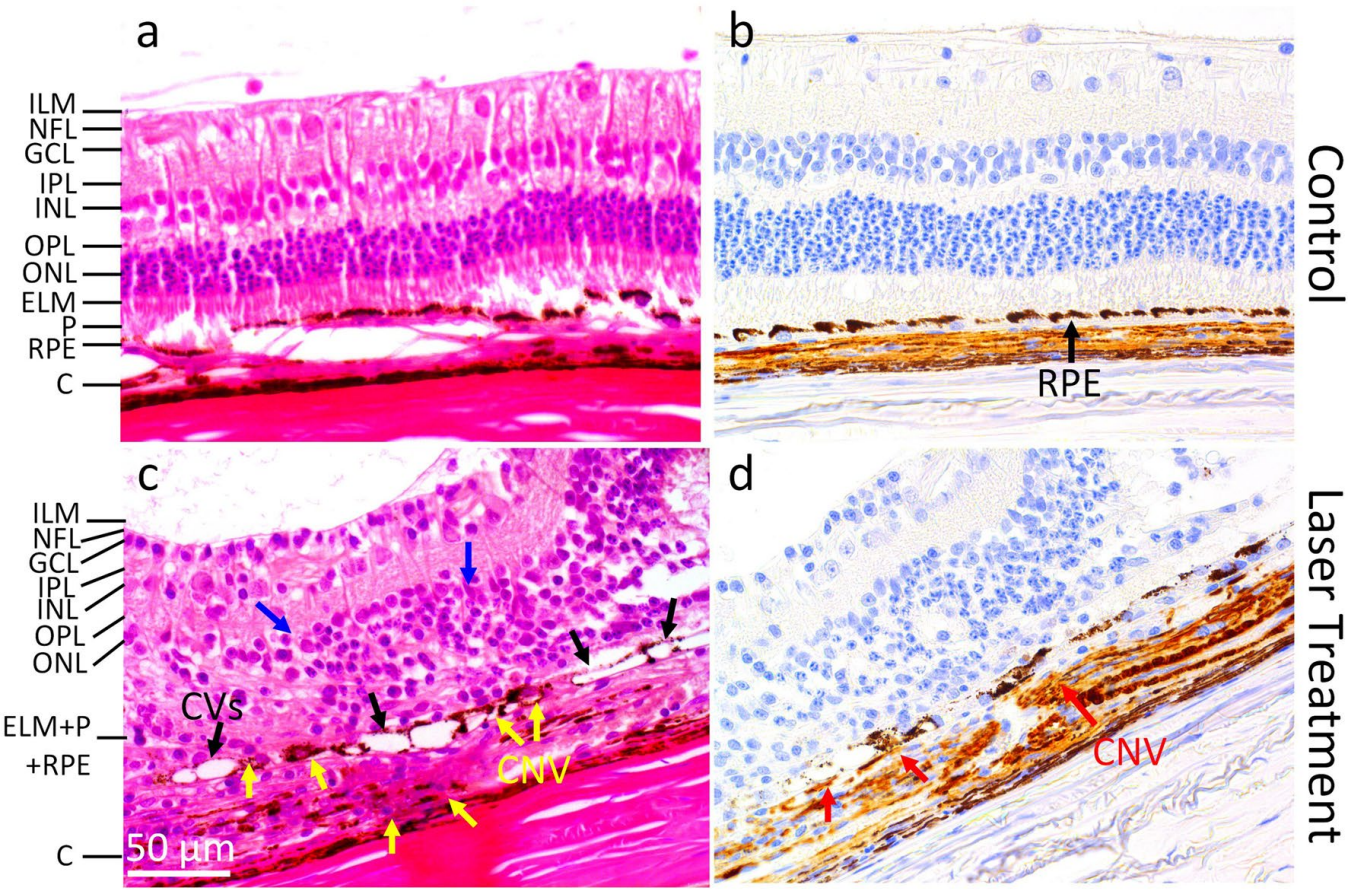
Histological and immunohistochemical analysis: (**a**) and (**c**) H&E staining images of control and treated groups, respectively. The H&E staining of the control group shows major retinal layers including the internal limiting membrane (ILM), nerve fiber layer (NFL), ganglion cell layer (GCL), inner plexiform layer (IPL), inner nuclear layer (INL), outer plexiform layer (OPL), external limiting membrane (ELM), photoreceptors (P), retinal pigment epithelium (RPE), and choroid layer (C). The retinal architecture and cells display normal morphology. In the treated group, there were significant changes in the retinal architecture (blue arrows). Scar tissue and cells were found in the treated area along with the development of neovascularization (yellow arrows). Black arrows indicate the location of choroidal vessels. Note that no retinal detachment was seen at the treated lesion. (**b**) and (**d**) corresponding immunostaining images with alpha SMA antibody of control and treated tissues. A monolayer of RPE was seen in the control group. In the treatment group, the RPE layer was discontinuous and CNV grew from the choroid layer and invaded the RPE layer and subretinal space through ruptured Bruch’s membrane (red arrows).

After 4 months, all the animals were euthanized for histological and immunohistochemical analysis to visualize the angiogenesis response in the treated eyes. The tissues were stained with H&E and alpha SMA antibody in order to visualize the development of CNV. Figure 4a shows the H&E image of control group without laser treatment. This image demonstrates different retinal layers including internal limiting membrane (ILM), nerve fiber layer (NFL), ganglion cell layer (GCL), inner plexiform layer (IPL), inner nuclear layer (INL), outer plexiform layer (OPL), outer nuclear layer (ONL), ellipsoid layer, photoreceptor (P), retinal pigment epithelium (RPE), and choroid layer (C). Figure 4b demonstrates the immunostaining image with alpha SMA antibody obtained from the control tissues without laser treatment. The RPE layer was clearly observed as a monolayer with melanin pigmentation. All the retinal layers are intact without any change in morphology or architecture. No new blood vessels were observed in the control group as well. After laser treatment, the retinal structure was significantly changed as shown in Fig. 4c–d. The retinal architecture was significantly changed and distorted, but there was no retinal detachment observed in the treated tissues. However, RPE, outer nuclear layer, photoreceptors, Bruch's membrane, and choroid layers at the treated areas were damaged and infiltrated with fibroblast tissue (Fig. 4c). H&E staining revealed neovascularization in all treated eyes. Immunohistochemistry with alpha-SMA confirmed that CNV developed from choroidal vessels, spread through the laser lesion region, and then grew into the subretinal space at the locations of injury found in H&E staining (Fig. 4d). These results demonstrate that H&E staining and immunohistochemistry with alpha-SMA can identify the formation of new blood vessels in subretinal lesions in pigmented rabbits.

## Discussion

Investigation on the pathophysiology in animal models are fundamental in order to discover and examine new therapeutic methods for various retinal disorders in humans. This study describes the first method to induce CNV in rabbits using laser photocoagulation with 100% success rate. The injured sites produced by laser photocoagulation were characterized by retinal destruction, rupture of Bruch’s membrane, and neovascularization growing from the choroidal layer, which is consistent with previous studies on laser-induced CNV models in other species such as mice and rats^17,27,39–41^. An important feature of this new clinically-relevant model is the persistence of CNV lesion in a large animal which has the axial eyeball length similar to that of the human eye. In addition, the circulation and pathology of rabbit retina are quite similar to that of the human eye, although the rabbit eye does not have a fovea or holangiotic retinal vasculature. As shown in Fig. 2, leaky CNV lesions were formed within the lesion area without affecting the adjacent retinal tissue and demonstrated stability for up to 4 months. This is a significantly longer follow-up duration than observed in our previous studies and in many other studies^10,12,22,31^. A longer follow-up CNV model in pigmented rabbits allows for a more comprehensive evaluation of the potential treatment's efficacy, safety, and long-term effects, which can inform future studies and the development of new therapies for neovascular AMD. In previous studies, we have demonstrated that CNV can be created by subretinal injection of Matrigel and VEGF. Although this method induced CNV similar to that observed clinically in AMD^10,15,33^, retinal structure and thickness were significantly affected and reduced with atrophy over the region of subretinal injection. Some retinal layers such as ONL, IPL, INL, OPL, and photoreceptors almost disappeared, and the retinal thickness was significantly reduced around the injected area^10^. Another advantage of the current study is that we developed a new laser protocol that uses a lower energy level to induce more consistent and reproducible CNV lesions with a defined size and morphology. The lifespan of the CNV lesions were evaluated by monitoring their size and fluorescence intensity over time and showed that the lesions persisted for at least 120 days.

We also demonstrate the potential of multimodal PAM, OCT, and fluorescence imaging for visualization of newly developed CNV and RPE and compared our technique with other conventional multimodal imaging systems for tracking CNV in animal models. In this study, we have successfully imaged CNV lesions created by retinal photocoagulation in rabbits using PA imaging. With the assistance of NIR contrast agents such as ICG dyes, PAM imaging has the ability to discern CNV from the surrounding RPE and retina vessels (Fig. 4b) by using different optical excitation wavelengths. For example, we used the excitation wavelength of 578 nm to reconstruct the morphology of both retinal vessels, RPE tissues, and new CNV lesions. By switching the excitation wavelength to 700 nm, one can only detect the margin of CNV because hemoglobin has very low absorption at this wavelength. This allows one to precisely distinguish CNV lesions. A limitation of the current study is that the PA imaging was acquired from day 30 post CNV development. Thus, the early stage of CNV progression were not completely monitored with PA imaging and compared to FA images. Therefore, early imaging of CNV formation after laser photocoagulation could be achieved with PA imaging in order to find the optimal treatment for CNV.

Another benefit of our system is that it combines anatomical information of retinal structure and retinal lesions provided by OCT imaging and correlates imaging with histological evaluation. We were able to precisely monitor the CNV progression over time. OCT provides the location of CNV and CNV thickness. But OCT has limited ability to visualize the degree of CNV as well as the growth of CNV within the treated area. In contrast, PAM imaging can provide this information. More importantly, PAM can differentiate CNV using multiple wavelengths that cannot be achieved by other optical imaging techniques. Thus, the combination of PAM and OCT allow the visualization of different CNV degree and size.

In conclusion, this study illustrates evidence that multimodal PAM and OCT is a potential technique for in vivo monitoring of a reproducible model of laser photocoagulation induced CNV in pigmented rabbits. This study provides comprehensive information of CNV lesions from OCT and PAM imaging with histological and immunohistochemical correlation to better understand CNV pathogenesis. These results reveal that this technique could be a useful approach in future studies of CNV diagnosis and monitoring.

## Materials and methods

### Animal model preparation

All animal studies performed in accordance with the ARRIVE guidelines and the ARVO (The Association for Research in Vision and Ophthalmology) Statement for the Use of Laboratory Animals in Ophthalmic and Vision Research. The procedures were implemented in accordance with the relevant guidelines and regulations and the study was approved by the Institutional Animal Care & Use Committee (IACUC) of the University of Michigan (Protocol PRO00010388).

Twelve Dutch Belted pigmented rabbits of both genders ages 2–4 months and weighing 1.9–3.0 kg were acquired from the Center for Advanced Models and Translational Sciences and Therapeutics (CAMTraST) at the University of Michigan Medical School. All of the rabbits in the study received choroidal neovascularization (CNV) by laser photocoagulation. The rabbits were housed in an air-conditioned room with a 12-h light–dark cycle, given free access to water, and administered standard laboratory food.

All rabbits were anesthetized intramuscularly with ketamine (40 mg/kg, 100 mg/mL) (JHP Pharmaceuticals, Rochester, MI, USA) and xylazine (5 mg/kg, 100 mg/mL) (Anased^®^, Boise, ID, USA). Tropicamide 1% ophthalmic and phenylephrine hydrochloride 2.5% ophthalmic were next administered to dilate the pupils. For topical anesthesia, 0.5% topical tetracaine or proparacaine was given. Phosphate-buffered saline (BRL, Life Technologies; Grand Island, NY, USA) was given every minute to maintain corneal hydration. Rabbit vital signs were monitored before, during, and after anesthesia, assessing the mucous membrane color, temperature, heart rate, respiratory rate, and oxygen saturation using a pulse oximeter (V8400D Capnograph & SpO2 Digital Pulse Oximetry, Smiths Medical, MN, USA).

### Dual-modality imaging equipment

**Figure 5:**
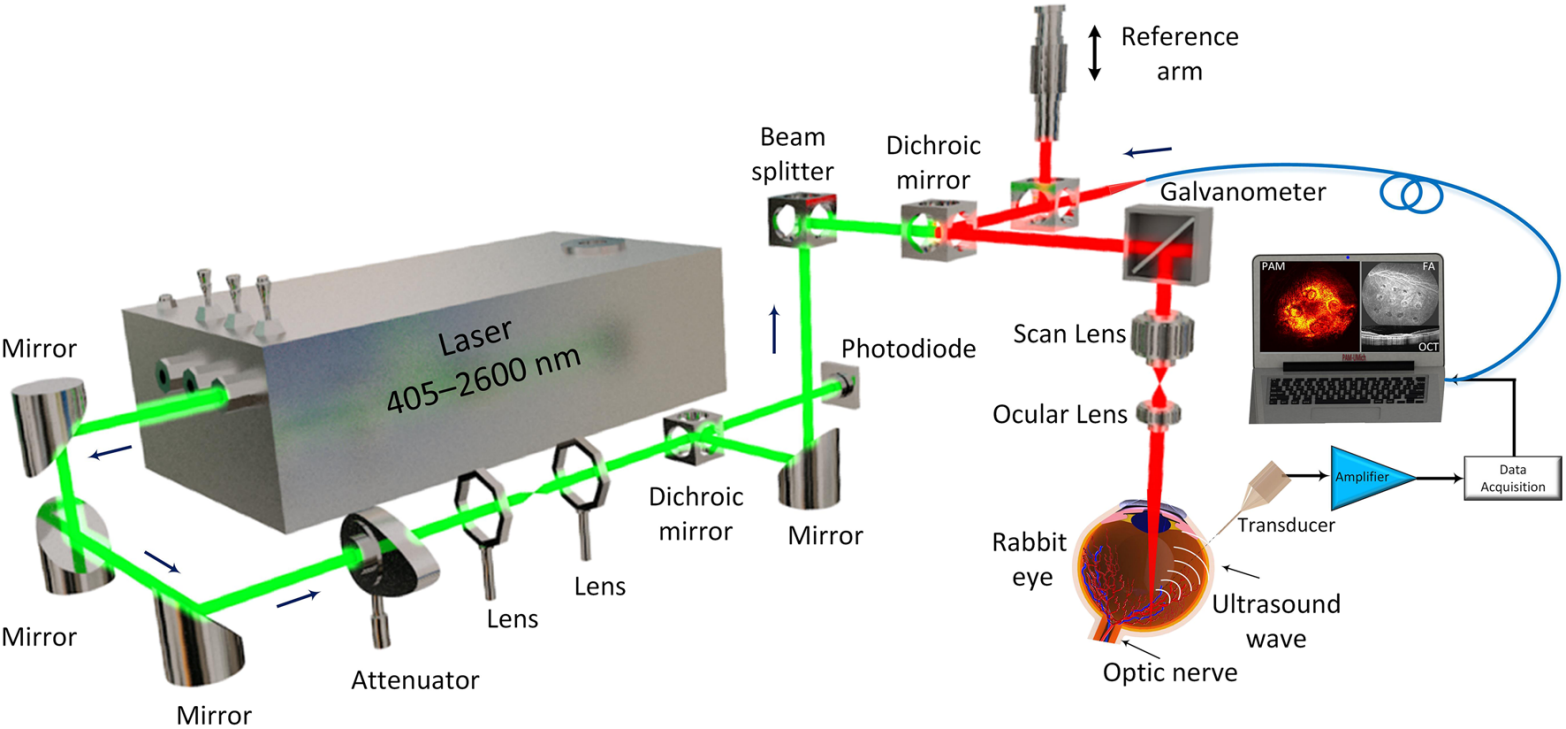
Schematic of the multimodal photoacoustic microscopy (PAM) and optical coherence tomography (OCT) system.

Figure 5 demonstrates the dual-modality imaging system that utilizes spectral-domain OCT (SD-OCT) and photoacoustic microscopy (PAM). Tian et al*.* have previously described the details of the system^14,20,38^. OCT is beneficial in detecting backscattered photons by low-coherence interferometry, while PAM provides high resolution and high depth of penetration. The SD-OCT system in the study was modified from a commercially available system from Thorlabs (Ganymede-II-HR, Thorlabs, Newton, NJ, USA). Briefly, an ocular lens was added to the system following an additional scan lens, and a dispersion compensation glass (DCG) was added to the OCT reference arm. The illumination source for PAM was an optical parametric oscillator (OPO) (NT-242, Ekspla, pulse duration 3–6 ns, wavelength tunable from 405 to 2600 nm). Endogenous chromophores like hemoglobin and melanin produced a PA signal detected by a custom-built needle-shaped ultrasonic transducer with a center frequency of 27.0 MHz (Optosonic Inc, Arcadia, CA, USA), which was directly in contact with PBS on the rabbit conjunctiva. This signal was digitized using a high-speed digitizer with a sampling rate of 200 MS/s (PX1500-4, Signatec Inc., Newport Beach, CA, USA) and amplified using a low-noise amplifier (gain 57 dB, AU-1647, L3 Narda-MITEQ, NY, USA). The PAM and SD-OCT have a lateral resolution of 4.1 μm and 3.8 μm, respectively, and the system can achieve an imaging depth of 1.9 mm. The OCT system’s center laser wavelength was 905 nm through two superluminescent light-emitting diodes with a center wavelength of 845 nm and 932 nm. The laser energy was half of the ANSI safety limit at approximately 80 nJ.

### Choroidal neovascularization (CNV) model

The choroidal neovascularization model (CNV) was implemented using laser photocoagulation on pigmented rabbits. First, a contact lens (Volk H-R Wide Field, laser spot 2 × magnification, Volk Optical Inc., Mentor, OH, USA) was placed directly on the cornea of the eye with Gonak Hypromellose Ophthalmic Demulcent Solution 2.5% (Akorn, Lake Forest, IL, USA). This solution provided coupling between the incident laser light and the cornea. The rabbit eye was then irradiated at a power of 300 mW with a solid state frequency-doubled Nd:YAG emitting at 532 nm green laser light (Vitra 532 nm, Quantel Medical, Cournon d’Auvergne, France) using a Zeiss SL 130 slit lamp (Carl Zeiss Meditec, Jena, Germany) attached to the Vitra photocoagulator with an adapter. The rabbit choroid was irradiated 16 times in a 4 × 4 square to demonstrate potential differences in CNV with increasing distance from the optic nerve and main retinal vessels (the medullary ray). The laser spot size was 500 µm in aerial diameter, and the pulse duration was 100 ms per spot. To determine the optimal laser power for laser induced CNV in pigmented rabbits, we evaluated the literature on the relationship of laser parameters and lesion characteristics^42,43^ and empirically treated the rabbit eyes with different laser powers such as 250 mW, 275 mW, 300 mW, 325 mW, and 350 mW along with the same laser spot size of 500 µm in aerial diameter, and the pulse duration of 100 ms. We found that there was no CNV observed on the eye treated with laser at 250–275 mW. In contrast, the choroidal vessels had significant hemorrhage under laser at high laser power of 350 mW (See the supplementary Fig. S3). Thus, the optimal laser power for CNV model was determined to be 300–325 mW.

### In vivo fundus photography, fluorescein angiography (FA), and indocyanine green angiography (ICGA) imaging

All rabbits were imaged with fundus photography, fluorescein angiography (FA), indocyanine green angiography (ICGA), PAM, and OCT both before and longitudinally up to 120 days after CNV generation with serial imaging. A pediatric Barraquer wire speculum was placed to open the eyelids. Fundus imaging was acquired using the Topcon 50 EX system (TRC 50EX, Topcon Corporation, Tokyo, Japan). FA imaging was completed by injecting 0.2 mL fluorescein sodium (Akorn, Lake Forest, IL, USA) solution intravenously in the marginal ear vein of the anesthetized rabbit. ICGA imaging was acquired by injecting 0.2 mL ICG dye (HUB Pharmaceuticals LLC, Patheon, Italy). The FA and ICGA contrast were imaged each minute for 5–15 min post-injection to monitor fluorescence and leakage indicating neovascularization. The fluorescence intensity and surface areas change over time was computed by comparing values generated from ImageJ. Hyperfluorescent CNV lesions were determined using the free-hand application. Regions of interest (ROI) were selected at the location of CNV and make sure the entire margin of CNV was selected. Average fluorescence intensity and surface areas were determined by ImageJ. The background intensity noise was calculated as an average of several images from different time points and subtracted from the values obtained from regions of CNV.

### In vivo multimodal PAM and OCT imaging for CNV

An anesthetized rabbit head and body were placed on two stabilization platforms to obtain PAM and OCT images to reduce motion artifacts. A water-circulation blanket (TP-700, Stryker Corporation, Kalamazoo, MI) was placed underneath the body to maintain adequate temperature. The integrated charge-coupled device (CCD) camera provided visualization to target the region of interest (ROI) during in vivo experiments, and the reference arm was calibrated to enhance image quality. B-scan OCT images were first acquired with a resolution of 512 × 1024 A-lines and an acquisition rate of 36 kHz. The PAM system was added to the OCT system and the ultrasonic transducer for multimodality imaging and controlled by Matlab2019b (MathWorks, MA, USA). Three-dimensional volumetric PAM images were obtained through raster scanning with an optical scanning galvanometer and rendered by Amira software. To distinguish CNV from the surrounding retinal vessels, ICG dyes were intravenously injected into the rabbits. At 15 min post-injection, the PAM images were obtained at 578 nm and 700 nm. CNV area was determined by measuring the number of positive pixels that had a higher intensity than the background on the PAM images. The choroidal neovascularization thickness and laser injury depth was calculated using ImageJ.

### CNV thickness measurement

To determine the CNV thickness, we performed auto semi-image segmentation to extract the area of CNV (Supplementary Fig. S2). Then, the CNV thickness was determined as an average of 12 different locations along the segmented CNV.

### Photoacoustic signal and vessel density analysis

To evaluate the PA signal increase between background and the CNV, region of interest (ROI) analysis was performed. The ROI with approximately 6000 pixels was randomly selected at the location of CNV on the acquired imaged PAM images to measure the mean PA signal. To minimize the measurement error, twelve different areas were selected around the CNV obtained from 700 nm to determine the mean PAM signal and standard deviation for each sample as described and performed previously^44^ (See the supplementary Fig. S4). Average PA signal was determined for each rabbit. Similarly, the vessel density (VD) was determined by counting the number of pixels where the PA signal is higher than background from the entire PAM image. The relative VD was measured at each time point for up to 120 days.

### Histological and immunohistochemical analysis

Histological analysis was performed to determine CNV and ocular damage. The rabbits were euthanized 120 days post photocoagulation. This was performed by intravenously injecting euthanasia solution (Euthanasia, 0.22 mg/kg, 50 mg/mL, VetOne, ID, USA). The whole eyes were immersion-fixed with Davidson’s (Hartmann’s) fixative for 24 h. The fixed eyes were rinsed in PBS and dehydrated using graded alcohols. The anterior cap (cornea, iris, and lens) was removed, separated, and sections were dissected. 5 mm pieces were embedded in paraffin and sectioned into 4 µm thick sections using Leica Autostainer XL (Leica Biosystems, Nussloch, Germany) and stained with hematoxylin and eosin (H&E). To further determine the development of CNV, immunohistochemistry was performed using anti-alpha smooth muscle actin (α-SMA) antibody to stain smooth muscle cells in vessel walls (Abcam, Burlingame, CA, US)^14^. These sections were analyzed using the Leica DM6000 light microscope (Leica Biosystems, Nussloch, Germany).

## Supplementary Information

Supplementary Information 1.

Supplementary Video 1.

## Data Availability

The data that support the plots and other findings of this study are available from the corresponding authors upon reasonable request.
