# Supplementary material for: Multimodal imaging of laser-induced choroidal neovascularization in pigmented rabbits
Source: Sci Rep. 2023 May 24;13:8396. doi: 10.1038/s41598-023-35394-z (PMC10209159; doi:10.1038/s41598-023-35394-z)
Supplement: Supplementary file 1 — Supplementary Information 1. [file 41598_2023_35394_MOESM1_ESM.docx]

Supplementary Information

Title: Multimodal Imaging of Laser-Induced Choroidal Neovascularization in Pigmented Rabbits

**Authors:** Van Phuc Nguyen^1^, Jessica Henry^1^, Josh Zhe^1^, Justin Hu^1^, Xueding Wang^2^, and Yannis M. Paulus^1,2*^

**Affiliations:**

^1^Department of Ophthalmology and Visual Sciences, University of Michigan, Ann Arbor, MI 48105, USA

^2^Department of Biomedical Engineering, University of Michigan, Ann Arbor, MI 48105, USA

^*^Corresponding Author:

Yannis M. Paulus, M.D., F.A.C.S.

Department of Ophthalmology and Visual Sciences

Department of Biomedical Engineering

University of Michigan

1000 Wall Street

Ann Arbor, MI 48105, USA

Email Address: ypaulus@med.umich.edu

**SUPPLEMENTARY FIGURES**

**
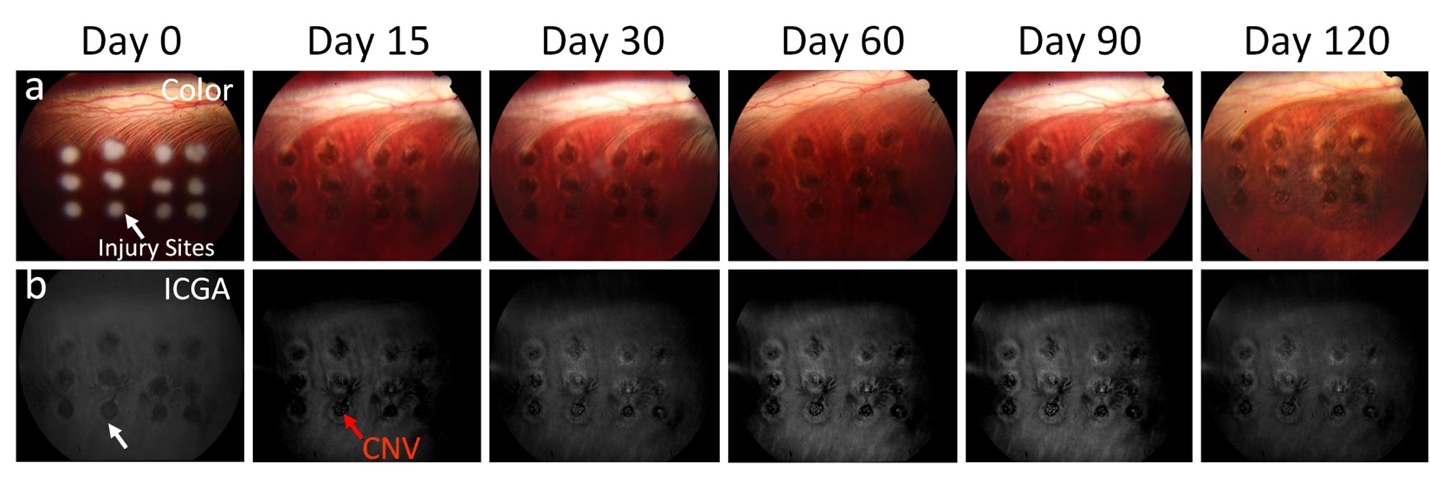
**

**Figure S1. Indocyanine green angiography (ICGA) imaging of CNV.** (a) Color fundus photography obtained at day 0 post laser treatment and day 15, 30, 60, 90, and 120 post treatment. (b) Corresponding late phase ICGA images acquired after intravenous injection of ICG dye at a dose of 25 μL and a final concentration of 2.5 mg/mL.


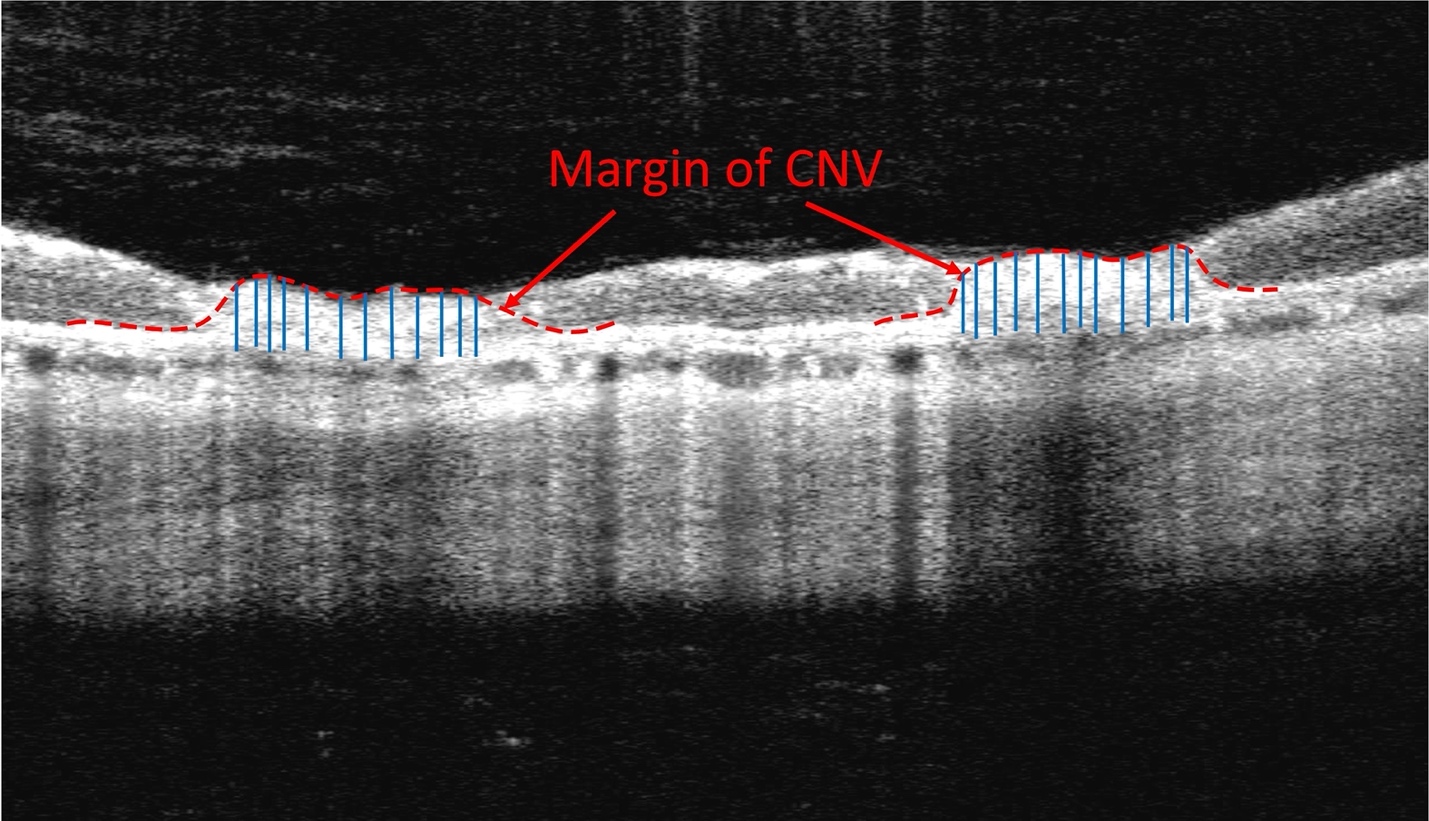


**Figure S2. OCT thickness measurement.** The margin of CNV was segmented using ImageJ (red dotted lines). Vertical blue lines indicate the selected region of interests (ROIs) to measure the thickness of CNV.

**
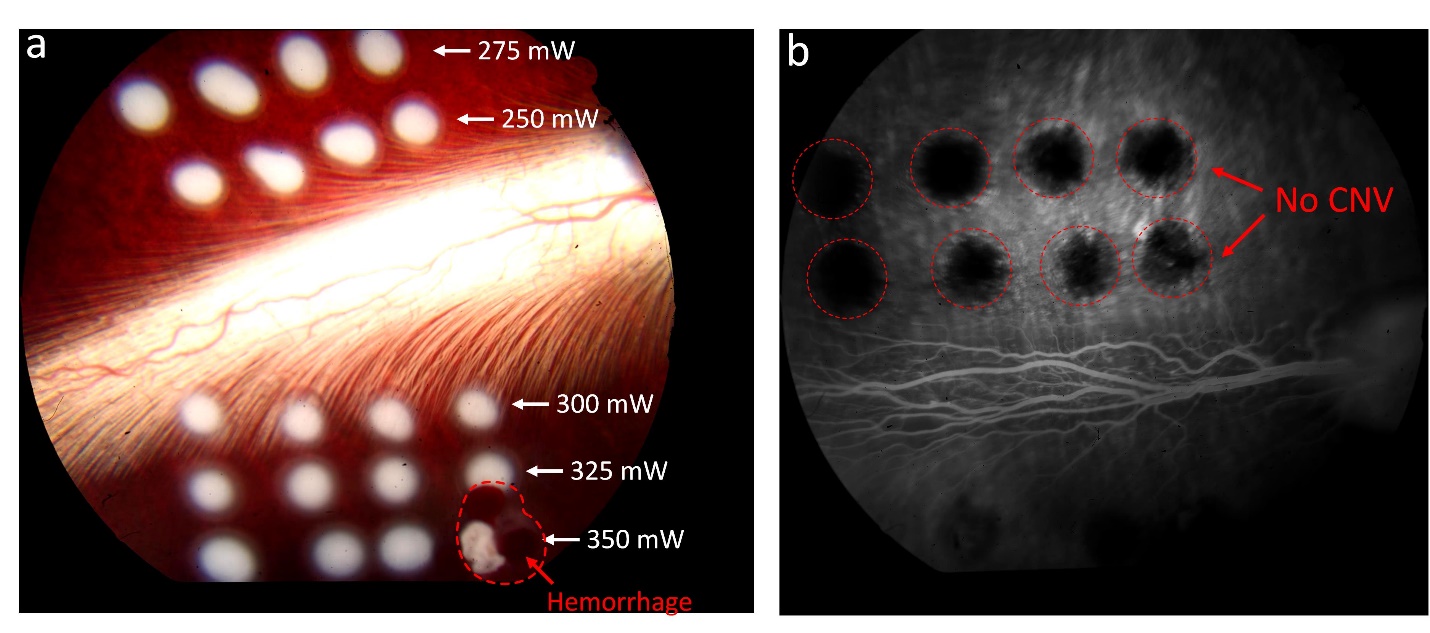
**

**Figure S3.** Laser power threshold evaluation for laser disruption of RPE layer. (a) Color fundus photograph of the retina after illuminated with laser at different laser powers: 250 mW, 275 mW, 300 mW, 325 mW, and 350 mW along with the same laser spot size of 500 µm in aerial diameter and pulse duration of 100 ms. Hemorrhage was found at the high power treatment at 350 mW (red dotted circle). (b) Late phase FA image obtained at 2-month post laser treatment. There was no evidence of CNV development found at the treated areas using 250 and 275 mW (red arrows).


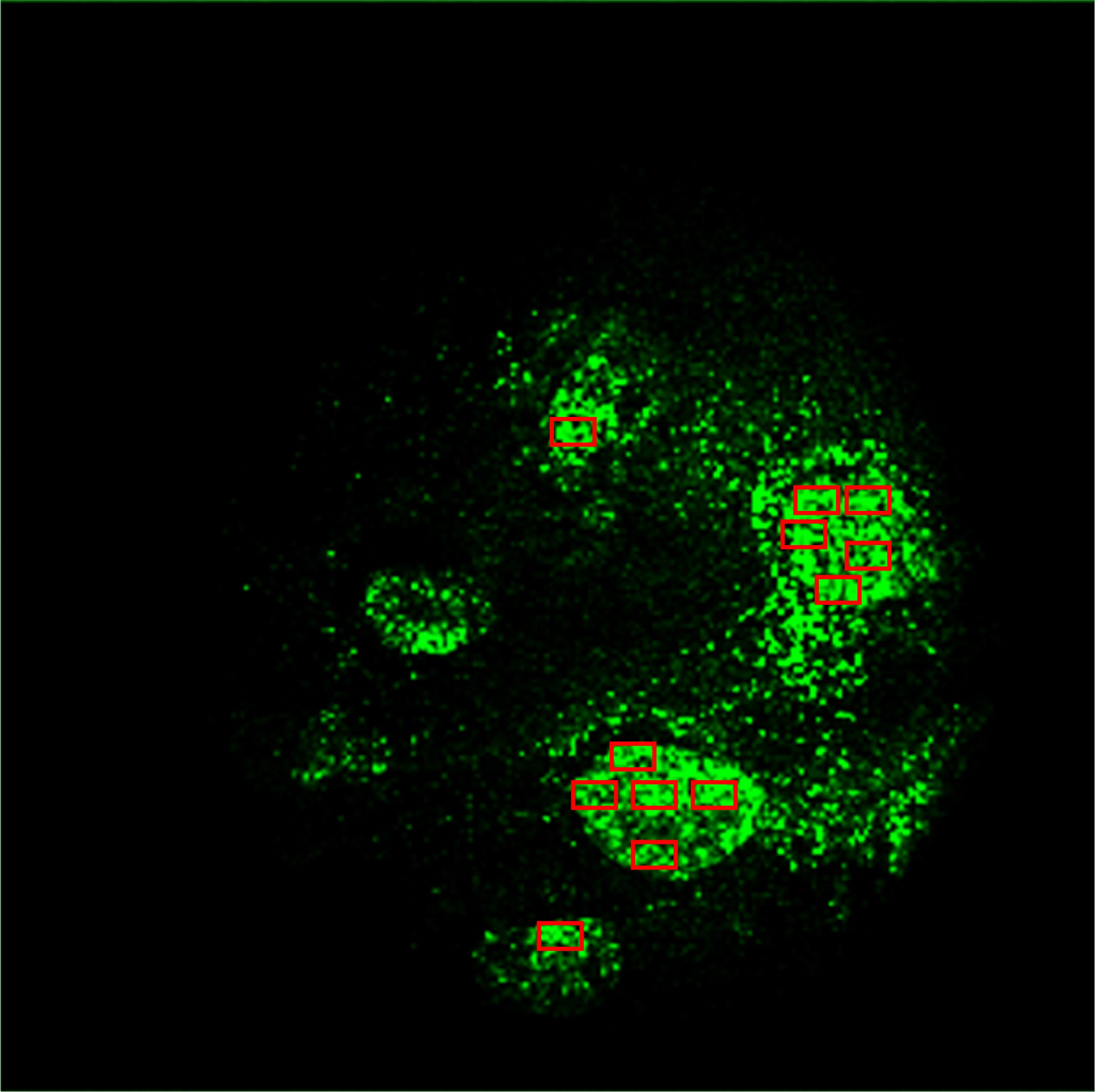


**Figure S4.** **PA signals and vessels density measurement.** Twelve different areas (red rectangles) were selected at the location of CNV on the PAM image acquired at 700 nm. Average signal was determined using the measurement function from ImageJ. The vessel density was determined by calculating the number of pixels which have signal stronger than that of the background.

**SUPPLEMENTARY MOVIE CAPTIONS**

**Movie S1.** Animated representation of 3D volumetric rendered PAM images is shown in Figure 3.
